# De Garengeot's hernia in a 60-year-old woman: a case report

**DOI:** 10.1186/1752-1947-5-258

**Published:** 2011-06-30

**Authors:** Petros Konofaos, Eleftherios Spartalis, Anastasios Smirnis, Konstantinos Kontzoglou, Grigorios Kouraklis

**Affiliations:** 1 2nd Department of Propedeutic Surgery, 'LAIKO' General Hospital, 36, Megistis Str, Athens 11364, Greece

## Abstract

**Introduction:**

De Garengeot first described the presence of the appendix within a femoral hernia in 1731.

**Case presentation:**

We report the case of a 66-year-old Caucasian woman who presented with acute appendicitis within an incarcerated femoral hernia. This is the first reported case of de Garengeot's hernia in the Balkan area.

**Conclusions:**

Appropriate management without incurring any delay for radiological imaging can be promising for an uneventful postoperative course. The treatment of choice of this disease entity is emergency surgery and consists in simultaneous appendectomy through the hernia incision and primary hernia repair. In patients with large hernia defects or in older people the use of mesh for repairing the hernia defect can be an excellent choice.

## Introduction

From 1731, when Rene Jacques Croissant de Garengeot first described the presence of the appendix within a femoral hernia [1], to date there have been fewer than 90 cases reported in the literature. de Garengeot's hernia is an incidental finding occurring in 0.9% of femoral hernia repairs [2], and appendicitis is rarer still, with an incidence of 0.08-0.13% [3]. There is a female predisposition (13:1, 93% in women), probably in keeping with the increased incidence of femoral hernia in women [3]. We report the case of a female patient with acute appendicitis within an incarcerated femoral hernia. This is the first reported case of de Garengeot's hernia in the Balkan area.

## Case presentation

A previously healthy 66-year-old Caucasian woman presented with a 24-hour history of sudden onset painful right-sided groin swelling. On clinical examination, there was a fixed, round, tender mass about 5 × 3 cm in size in the right groin, above the inguinal crease. Her temperature was 38.7°C and she did not appear to be in distress. She did not have any bowel obstruction revealed by clinical examination or on the abdominal X-ray. Her past medical history was insignificant. Her laboratory findings were within normal limits except an increased WBC count (13.00 K/μL) with 80% neutrophils.

**Figure 1:**
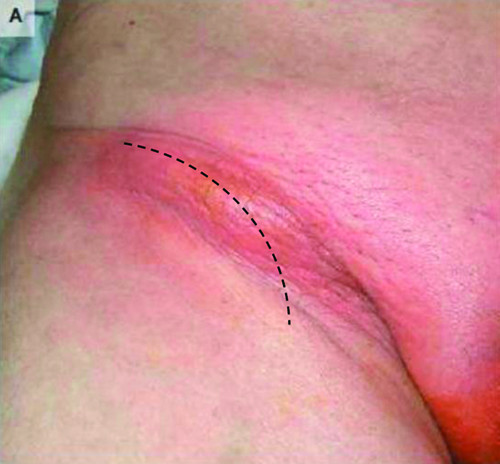
**Preoperative frontal view that demonstrates a red, round bulge in the groin area**. The black dotted line shows how the curved low inguinal incision was performed

**Figure 2:**
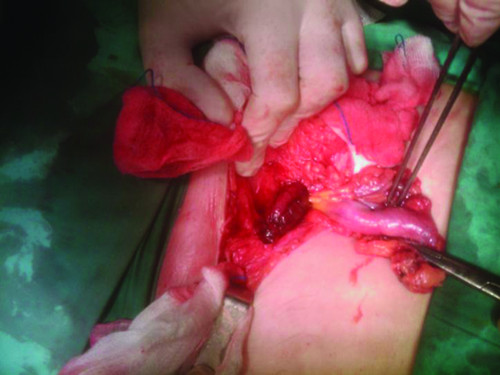
**Intraoperative image of the inflamed gangrenous appendix protruding through the femoral hernial sac**.

A presumptive diagnosis of a chronically incarcerated femoral or inguinal hernia versus a strangulated hernia or an inguinal abscess was made with plans for a right groin exploration using a more curved low inguinal incision under general anesthesia (Figure 1). When the hernia sac was opened, an inflamed appendix was seen. The appendix was thickened and inflamed, but there was no perforation. Intraoperative findings were consistent with an inflamed and gangrenous appendix protruding through the femoral hernial sac (Figure 2). Routine appendectomy was performed through the hernial sac. The mouth of the hernia was wide and the senior surgeon was even able to pass a finger through the hernia into the peritoneal cavity. The hernial sac was closed using a V-shaped polypropylene mesh. A broad-spectrum antibiotic cover was provided at induction. The postoperative course was uneventful and the patient was discharged home on the third day after the procedure. The histological examination was consistent with acute appendicitis.

## Discussion

Although femoral hernias account for 4% of all groin hernias, a hernia sac can contain any of the intraabdominal contents such as omentum. A pelvic appendix has the highest risk of entering a femoral hernial sac [4]. The evolution of inflammation in the appendix is thought to be secondary to its engagement in the hernial sac. Although there are occasional cases diagnosed preoperatively, typically the appendix is found incidentally during repair without any preoperative signs or symptoms [5].

De Garengeot's hernia is often misdiagnosed as an incarcerated or strangulated femoral hernia. The incidence of an appendix in a femoral hernia is reported to be 0.5-5% [2,6-8]; the reason for this wide variation is the paucity of cases and no published large case series. The clinical picture of this entity is that of incarcerated femoral or inguinal hernia and includes vague abdominal pain and tenderness and an erythematous groin lump [7]. The signs of appendicitis are overshadowed by a tight femoral hernia neck and pelvic rigidity; this anatomical feature prevents the spread of inflammation to the peritoneal cavity [9].

Abdominal X-ray does not aid in the diagnosis of de Garengeot's hernia. Computed tomography (CT) and ultrasound have been successfully used for preoperative evaluation [10]. The presence of a low-positioned cecum along with tubular structure within the hernial sac and stranding of nearby fat on CT have been reported to have 98% specificity and sensitivity for diagnosing or ruling out appendicitis within a hernial sac. In our case, further preoperative radiological refinement (with either CT and/or ultrasound) would not have changed the decision to operate as this patient had a clinically strangulated hernia,

The treatment of choice of this disease entity is emergency surgery [6] and consists in simultaneous appendectomy through the hernia incision and primary hernia repair. Although alternative approaches have been described in the literature, the low curved inguinal approach adopted in this case provided adequate exposure for both the femoral canal exploration and intraabdominal access. Alternative approaches such as Cooper's ligament repair and a preperitoneal approach [6] have been described in the literature, but the low inguinal approach adopted in this case provided adequate exposure for both the femoral canal exploration and intraabdominal access.

Choice of repair in a femoral hernia containing a pathological appendix is debatable. Generally prosthetic material is not preferred in a contaminated field due to the risk of infection [10], but a few reports have mentioned mesh repair even in the presence of an inflamed appendix with no postoperative infection [11].

Even though there is at least one report of infection with the use of mesh, even in the absence of acute appendicitis [6], this reconstructive option has to be adopted by the surgeon especially in cases with large hernia defects or in older patients (in order to avoid hernia recurrence). The presence of perforation of the appendix is a contraindication for the use of mesh for repairing the hernia defect. In recent studies, the consensus is that if there are no signs of abscess formation or perforation, repair by prosthetic mesh is possible without infection or recurrence [12]. Nguyen et al [13] pointed out that the factor contributing to the increased incidence of infection is the delay in diagnosis.

In this case, the operation was performed immediately and no abscess was found in the hernial sac. There was no evidence of perforation and the patient was more than 60-years-old.

The most common complication of the de Garengeot's hernia repair is wound infection with a rate reaching 29%. Some cases of necrotizing fasciitis and even death have been reported [5], probably related to the delay in diagnosis and the older age of the patients.

## Conclusions

Although the incidence of de Garengeot's hernia is extremely low, the surgeon has always to keep it in mind in cases with femoral hernias and regional symptoms of inflammation due to the lack of abdominal signs of appendicitis. Appropriate management without incurring any delay for radiological imaging can be promising of an uneventful postoperative course. In patients with large hernia defects or in older patients the use of mesh for repairing the hernia defect can be an excellent choice.

## Consent

Written informed consent was obtained from the patient for publication of this case report and any accompanying images. A copy of the written consent is available for review by the Editor-in-Chief of this journal.

## Competing interests

The authors declare that they have no competing interests.

## Authors' contributions

All authors read and approved the final manuscript. PK was a major contributor in writing the manuscript. ES was involved in acquisition of data and review of the literature. AS was involved in acquisition of data and review of the literature. KK was involved in drafting the manuscript and revising it critically for important intellectual content. GK was involved in drafting the manuscript, revising it critically for important intellectual content and gave final approval of the version to be published.
